# Resistance of a terrestrial plant community to local microhabitat changes

**DOI:** 10.1002/ece3.4093

**Published:** 2018-04-24

**Authors:** Jerrold M. Tubay, Jin Yoshimura

**Affiliations:** ^1^ Mathematics Division Institute of Mathematical Sciences and Physics University of Philippines Los Baños College Laguna Philippines; ^2^ Graduate School of Science and Technology Shizuoka University Hamamatsu Japan; ^3^ Department of Mathematical and Systems Engineering Shizuoka University Hamamatsu Japan; ^4^ Marine Biosystems Research Center Chiba University Uchiura, Kamogawa Chiba Japan; ^5^ Department of Environmental and Forest Biology State University of New York College of Environmental Science and Forestry Syracuse New York

**Keywords:** coexistence, dynamic microhabitat locality, lattice Lotka–Volterra model, modeling (community ecology), simulation

## Abstract

The number of plant and animal species that exist today is estimated to be around 8.7 million. Approximately 300,000 of these species are flora. This extremely high species diversity has been puzzling scientist since the beginning of ecological research because most of these species compete for limited resources that should lead to the exclusion of all but few superior species. This can be seen in a number of coexistence model today that can only maintain at most four species at a time. We have shown recently that by incorporating minute differences in microhabitat to a lattice competition model, about 13 species can coexist from an initial number of 20. Here, we improve the model further by considering that microhabitat differences are not fixed but can change over time which can affect coexistence. A primary driver to this alteration is climate change, both natural and human induced. To show the resistance of a lattice plant community model, a dynamic microhabitat locality is incorporated by changing the spatial and species‐specific heterogeneity of each lattice site. We show that even if the microhabitat locality of each plant species is dynamic, diversity can still be maintained in a lattice plant ecosystem model. This shows that natural communities of terrestrial plants can be resistant to the stress of microhabitat locality changes to a certain extent.

## 1 INTRODUCTION

Years have passed since Tilman suggested that minute differences in microhabitat can be the reason for coexistence (Tilman, 1988, 1992). For instance, two small patches of soil adjacent to each other in grassland and forest communities can be very different in terms of nutrient composition and moisture content (Kleb & Wilson, 1997; Shiyomi et al., 2001). This soil heterogeneity is brought about by many factors such as uneven animal excretion, the presence of animal carcass on some soil patches, irregular distribution of dead leaves and wood, microbial activity, sunlight, canopy cover, and topography (Kleb & Wilson, 1997). Even soils that are used for agriculture are never homogeneous (Adamchuk, Ferguson, & Hergert, 2010). However, micro‐spatial heterogeneity in soils has not been incorporated in previous models until recently (Tubay et al., 2015). These changes in microhabitats can be induced by an increase in temperature due to global warming but are generally applicable to yearly and gradual changes in microhabitats. Our focus here is to evaluate the effects of the common environmental variability of microhabitats on plant species diversity as it changes over time. In our previous paper, we showed that even minuscule differences in microhabitat composition can promote coexistence in a lattice competition model. This model is different from other coexistence models as the lattice model presented in our previous work can maintain stable coexistence of more than 10 species while most coexistence models are based on lottery models which can only maintain at most four species with strict stability requirements (Tubay et al., 2015). Nevertheless, we assumed in the previous model that the minute differences in microhabitat are fixed over time. In reality, spatial locality can change due to natural and human‐induced climate change. This means that after several or few generations, a patch of soil which is friendly to a specific species of flora may not be as welcoming in the future (Williams, Shoo, Isaac, Hoffmann, & Langham, 2008). Although this is the case, most plant ecosystems demonstrate resistance to environmental changes (Thompson, 2010). A specific example is an infertile grassland in northern England which is highly resistant to temperature and rainfall modification, a long‐term experiment on the effect of climate manipulation to plant diversity (Grime et al., 2008). Another study that shows plant resistance to a changing climate shows the drought resistance of several woody seedlings with little effect on their survival (Engelbrecht & Kursar, 2003).

Here, we test whether a dynamic microhabitat locality promotes coexistence of many species in a lattice terrestrial ecosystem model of competing sessile organisms. That is, a lattice plant competition model with microhabitat locality is resistant to change, at least to some extent. There are many previous models relating spatial heterogeneity to coexistence; however, these models only considered spatial heterogeneity as different location or habitat in space with respect to an individual species (Chesson & Warner, 1982, 2000; Muko & Iwasa, 2000; Takenaka, 2006). This difference in habitats in their models only implies differences in species fecundity and mortality. Moreover, most of their models are based on lottery models which lead to competitive exclusion in the long run. In this study, spatial heterogeneity means physical variations in microhabitat, not just the position in space. To represent the minute differences in microhabitat, we assigned settlement rates for each lattice site and species that represents germination or seedling success. To incorporate a dynamic spatial heterogeneity, these settlement rates are changed in the simulation at regular time intervals. First, we will assume that the changes are independent of each other (total change in microhabitats). In the model, as microhabitat locality is represented by random numbers between 0 and 1, a total change means a new set of random numbers. The second scenario that will be simulated is when the changes are dependent (partial change in microhabitats). Dependency here means that the new set of microhabitat localities is computed from the previous one, unlike the first scenario where new microhabitat localities were generated independently. The first scenario can model an environment that changes drastically over time while the second one replicates a gradual change in microhabitat locality depending on the percentage change. From these scenarios, we will show that although microhabitat locality is changing over time, a relatively stable coexistence of different species is still possible. In addition, we will also determine the circumstances by which coexistence is severely affected.

## 2 MATERIALS AND METHODS

### 2.1 Simulation overview

A lattice Lotka–Volterra model is used to examine the dynamics of a 20‐species system representing the plant ecosystem. Each species has a different basal fecundity measure similar to the original model with a fixed mortality rate for all species. Fecundity rates of species are computed in a decreasing manner from a maximum basal fecundity rate. Minute differences in microhabitats are represented by a random parameter whose value is in the interval which is different for each species and for each lattice site which represents a microhabitat. These microenvironmental differences are set to change in a regular manner, to mimic the effect of climate change and other natural or human activities that affect microhabitats such as climate change, human induced, or otherwise.

After thousands of simulations, we show that a significant number of species are generally plausible to coexist in a relatively small lattice space of 20 total species even with regular or random changes in microhabitat locality. This shows the resistance of coexistence even in the presence of factors that change microhabitats such as climate change. However, exclusion can happen if the rate of change in microhabitat is high. This coexistence dynamics can be applicable to temperate and tropical forests, grasslands, and other vegetation types that are threatened by the fast‐changing climate.

### 2.2 Lattice model

A lattice is a two‐dimensional system that represents the space occupied by a certain biological community (e.g., plant community) where the lattice sites represent the microhabitats. These microhabitats are small spaces where one and only one individual of a certain species of plant can occupy. In other words, the lattice system itself, although abstract, is a physical representation of an ecosystem in two dimensions. The simulations were conducted using a 200 × 200 lattice Lotka–Volterra competition model with competing sessile species *i* from 1 to *s*, where *s* is the total number of different species. This is a two‐dimensional lattice where plant species compete for space (i.e., direct sunlight and soil). Each of the square lattice sites can be occupied by one and only one individual of a plant species denoted by *X*_*i*_ while a vacant site is represented by *O*. The population dynamics among the species is based on multi‐species contact process defined by

(1) $$X_{i}+O\to X_{i}+X_{i}\;\text{at the rate of}\;b_{i}$$

(2) $$X_{i}\to O\;\text{at the rate of}\;m_{i}$$

where *b*_*i*_ and *m*_*i*_ are the effective birth rate and mortality rate of species *i*, respectively. The birth process is carried out using local and global interactions between pairs of lattice sites in separate simulations. In both interactions, a pair of random lattice sites is chosen, one occupied and the other is vacant. With the local interaction, the pair of lattice sites are four‐neighborhood adjacent while in the global interaction, the pair can be located separately anywhere in the lattice model.

In this model, the dynamics between individuals of different species compete for lattice space and this competition depends solely on the effective birth rates of each species and a fixed mortality rate similar to all species (Table 1).

**Table 1:** Parameters

Parameter	Description
L	Lattice dimension
L×L	Lattice size or carrying capacity
*s*	Number of different competing species
*i*	Index for a specific species
$X_{i}$	Label for a lattice site that is occupied by an individual of species *i*
O	Label for a vacant lattice site
$b_{i}$	Effective birth rate of species *i*
$B_{i}$	Basal fecundity of species *i*
*a*	Basal fecundity of the most superior species assigned to i=1
*r*	Minimum difference between fecundity among species
*p*	Upper bound for the fecundity difference between species
$m_{i}$	Mortality rate of species *i*
$\varepsilon_{i}\left[m,n\right]$	Microhabitat locality of species *i* at site $\left[m,n\right]$
$\varepsilon_{i}\left[m,n\right]\left(t\right)$	Microhabitat locality of species *i* at site $\left[m,n\right]$ at time t
α	Percent change in microhabitat locality for dependent or partial change scenario

As with the previous microhabitat locality studies we did, we introduced the site‐ and species‐specific variability parameter ε_*i*_[*m*,* n*] which is incorporated in the effective birth rate *b*_*i*_ of species *i*, that is

(3) $$b_{i}=B_{i}\cdot \varepsilon_{i}\left[m,n\right]$$

where *B*_*i*_ is the fecundity of species *i* and ε_*i*_ [*m*,* n*] is the random parameter representing the specificity of species *i* at a lattice site [*m*,* n*]. To reach the stable state of the populations quickly, variations between the basic fecundity *B*_*i*_ among species are introduced, where $B_{i}=a-\left(i-1\right)r$ for i=1,2,…,s. The parameter *a* is the fecundity of *B*_1_, the most superior species in terms of basic fecundity and minimum difference between fecundity among species is set to r=p/20, where *p* is the upper bound of the fecundity difference (Tubay et al., 2015).

With this model, *B*_*i*_ can be considered as species *i*'s birth rate without the environmental factors while $\varepsilon_{i}\left[m,n\right]$ is the local settlement rate of species *i* at a specific microhabitat [m,n]. The parameter $\varepsilon_{i}\left[m,n\right]$ follows a standard normal distribution over the interval [0,1]. If there is no species specificity, then $\varepsilon_{i}\left[m,n\right]=\varepsilon \left[m,n\right]$, while the lack of site specificity implies that $\varepsilon_{i}\left[m,n\right]=\varepsilon_{i}$.

In the real world, microhabitat differences are not static because of the changing environment. In this simulation model, $\varepsilon_{i}\left[m,n\right]$changes over time t in regular intervals, denoted by$\varepsilon_{i}\left[m,n\right]\left(t\right)$. That is,

(4) $$\begin{array}{cc} \varepsilon_{i}\left[m,n\right]\left(u\right) & =\varepsilon_{i}\left[m,n\right]\left(u+1\right)=\cdots =\varepsilon_{i}\left[m,n\right]\left(u+k-1\right)\\ & \;\neq \varepsilon_{i}\left[m,n\right]\left(u+k\right) \end{array}$$

where *u* is a positive integer divisible by *k*. These changes in microhabitat locality can represent the effect of climate change in natural plant communities (Bellard, Bertelsmeier, Leadley, Thuiller, & Courchamp, 2012). Microhabitat locality is changed in two different scenarios. The first scenario is when $\varepsilon_{i}\left[m,n\right]\left(u+k\right)$ is generated independently from the previous microhabitat locality $\varepsilon_{i}\left[m,n\right]\left(u\right)$. The other scenario is when the changes are dependent, that is,

(5) $$\varepsilon_{i}\left[m,n\right]\left(u+k\right)=\left\{\varepsilon_{i}\left[m,n\right]\left(u\right)\right\}\cdot \alpha +\left[rand\left(0,1\right)\right]\cdot \left(1-\alpha \right)$$

where 0 < α < 1 is the percent change in the microhabitat locality of species *i*.

### 2.3 Simulation model

The simulation procedure is as follows (for the computer simulation code written in C, please refer to the supporting information):

#### 2.3.1 Initialization

Individuals of plant species *i* are distributed randomly in the lattice of size *L* × *L* with an initial population density of *I*_*i*_ in such a way that each site is occupied by one and only one individual of a certain species. Also, the initial microhabitat locality $\varepsilon_{i}\left[m,n\right]\left(0\right)$ of species *i* at site $\left[m,n\right]$ is generated randomly for all species *i*. Set a positive integer ν that will indicate the times by which $\varepsilon_{i}\left[m,n\right]$ will change (e.g., 50,000 changes—every generation, 100 changes—every 500 generations, etc.).

#### 2.3.2 Iterative steps *k*

The birth and death processes (also called reaction process) are performed as follows:

1. *Birth Process*. To perform the birth process or the two‐body reaction (1), two lattice sites are chosen randomly. If the selected pair are *X*_*i*_ and *O*, respectively, the empty site *O* changes with the probability *b*_*i*_. Otherwise, the points remain unchanged. Note that we utilize periodic boundary conditions.
2. *Death Process*. Next, perform the single‐body reaction or the death process by choosing a single lattice square randomly. If the selected site is *X*_*i*_, then it is changed to *O* with probability *m*_*i*_. No change otherwise.
3. Repeat step *k*,* L *× *L* times which is the total number of lattice sites. If *k* is divisible by ν, we generate a new set of $\varepsilon_{i}\left[m,n\right]$.

#### 2.3.3 Stopping criterion

The simulation terminates after a specified number of generations and trials which are set to 50,000 and 50, respectively, for robustness of results.

## 3 RESULTS

### 3.1 Independent microhabitat locality changes

**Figure 1:**
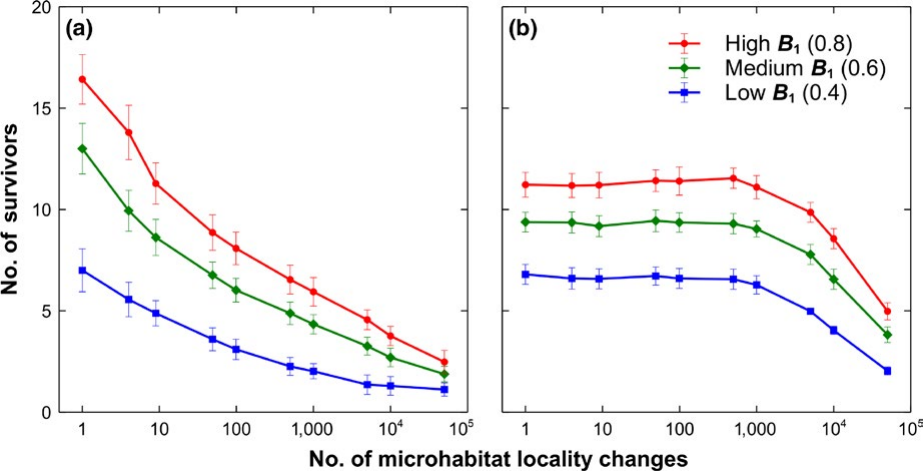
Coexistence and independent changes in microhabitats. Simulation results of a 20‐species lattice Lotka–Volterra competition model with independent microhabitat locality changes and different maximum basal fecundity $B_{1}$ (Average of 50 runs at 50,000 generations with the corresponding standard deviations as error bars). (a) Temporal changes in the number of surviving species with respect to the number of microhabitat changes under local dispersion. (b) Temporal changes in the number of surviving species with respect to the number of microhabitat changes under global dispersion

A comparison of the results between local and global interaction is shown in Figure 1. As expected, both interactions show a decreasing trend in the number of coexisting species as the number of changes in microhabitat locality increases for both local and global dispersion. This demonstrates that coping with a number microhabitat changes is stressful to the population if the changes are rapid. However, global dispersion shows less stress as compared to a local dispersion. Although the maximum number of coexisting species is smaller, it can be observed that the effect of the changes is only visible when the number of microhabitat changes exceeds 1,000 after 50,000 generations. This shows that coexistence is more resistant when plants are capable of dispersing anywhere in the community.

**Figure 2:**
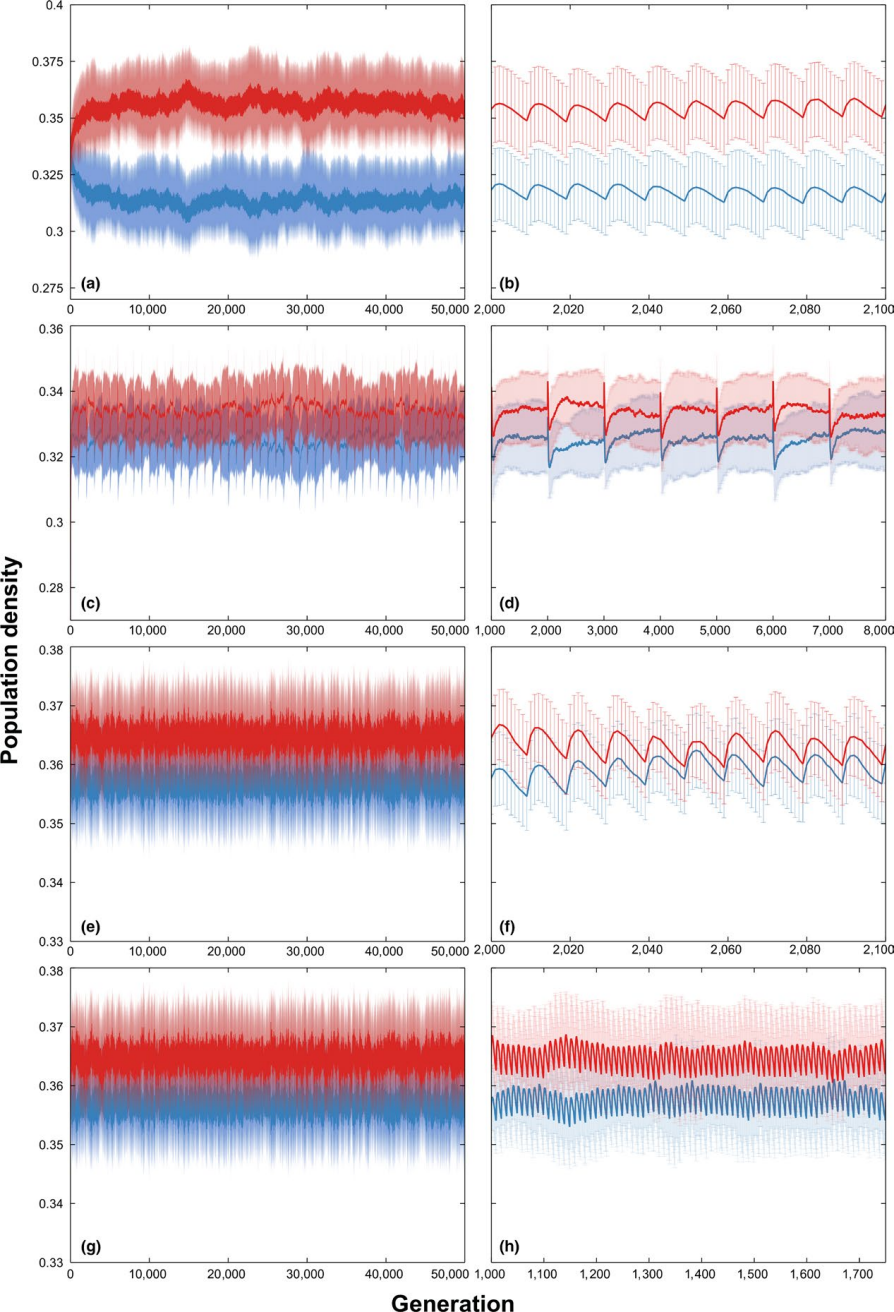
Two‐species dynamics with independent microhabitat changes. Simulation results of a two‐species lattice Lotka–Volterra competition model with independent microhabitat locality changes (Average of 50 runs at 50,000 generations with the corresponding standard deviations as error bars). (a) Average population dynamics and deviation of a two‐species system where microhabitat locality changes every 10 generations under global interaction. (b) Magnified plot of (a). (c) Average population dynamics and deviation of a two‐species system where microhabitat locality changes every 1,000 generations under global interaction. (d) Magnified plot of (c). (e) Average population dynamics and deviation of a two‐species system where microhabitat locality changes every 10 generations under local interaction. (f) Magnified plot of (e). (g) Average population dynamics and deviation of a two‐species system where microhabitat locality changes every 1,000 generations under local interaction. (g) Magnified plot of (f)

To examine the mechanism of coexistence in the lattice model with a dynamic microhabitat locality, a two‐species system is observed (see Figure 2). The dynamics of the populations of the two species can be clearly seen in the magnified plots on the right (see Figure 2B, D, F, H). Every time the microhabitat locality changes, the population fluctuates on the average. Although this might be the case, the fluctuations have no adverse effect on the coexistence of the two species. Generally, the coexistence of the two species regains its stability after the fluctuations even though the number of microhabitat changes is increased considerably.

### 3.2 Dependent microhabitat locality changes

Changes in the microhabitat locality of species for the next period can be dependent on its current value. Several instances where simulated depending on the scale of change in microhabitat locality and the speed of the said change. The scale of change can as small as 0.001% or as much as 90%. These instances were simulated to elucidate the effect of such parameters to coexistence in a lattice plant community. Unlike independent changes were (100% microhabitat locality change), simulating the different levels of changes can better elaborate on the effect of dynamic microhabitat locality to coexistence.

**Figure 3:**
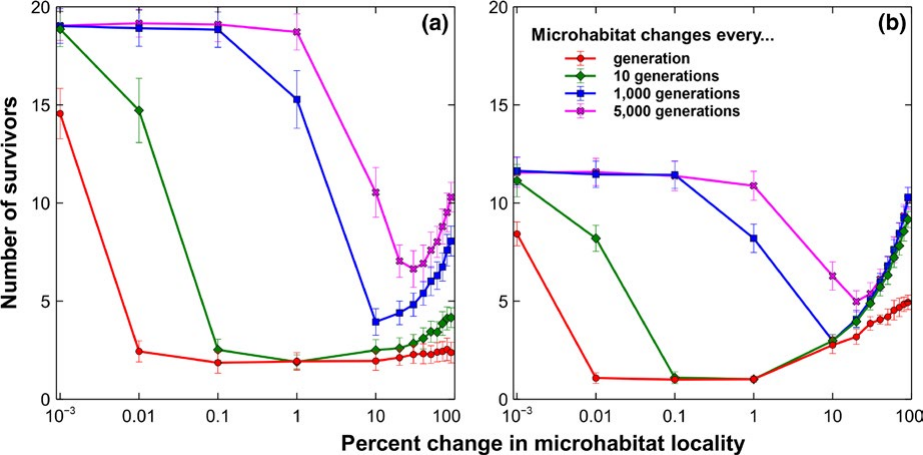
Coexistence and level of changes in microhabitats. Simulation results of a 20‐species lattice Lotka–Volterra competition model with dependent microhabitat locality changes (Average of 50 runs at 50,000 generations with the corresponding standard deviations as error bars). (a) Temporal changes in the number of surviving species with respect to the percentage change in microhabitat locality under local dispersion. (b) Temporal changes in the number of surviving species with respect to the percentage change in microhabitat locality under global dispersion

Figure 3 shows the summary of the simulations with dependent microhabitat locality changes for local and global dispersion. It can be clearly seen that when the changes are rapid, even minute changes in microhabitat locality have an adverse effect in coexistence. If microhabitat locality changes every generation, a mere 0.01% change can reduce the number of coexisting species from ~15 down to about three species for local dispersion (see Figure 3a). However, when a 1% change happens only every one thousand generations, the number of coexisting species is only reduced from approximately 19 species down to about 16 when the dispersion is local (see Figure 3a). This observation is also true for global seedling dispersion (see Figure 3b).

Remarkably, the number of coexisting species increases after a significant decrease caused by an increased percentage change in microhabitat locality. This observation can be seen in both local and global dispersion. Note that a 100% change in microhabitat locality is the same as the independent scenario (see Figure 1). Although there is an increasing trend in the number of coexisting species, the maximum number of coexisting species in this increase is still significantly smaller compared to a static microhabitat locality except for the global dispersion where microhabitat locality changes every one thousand generations (see Figure 3b).

**Figure 4:**
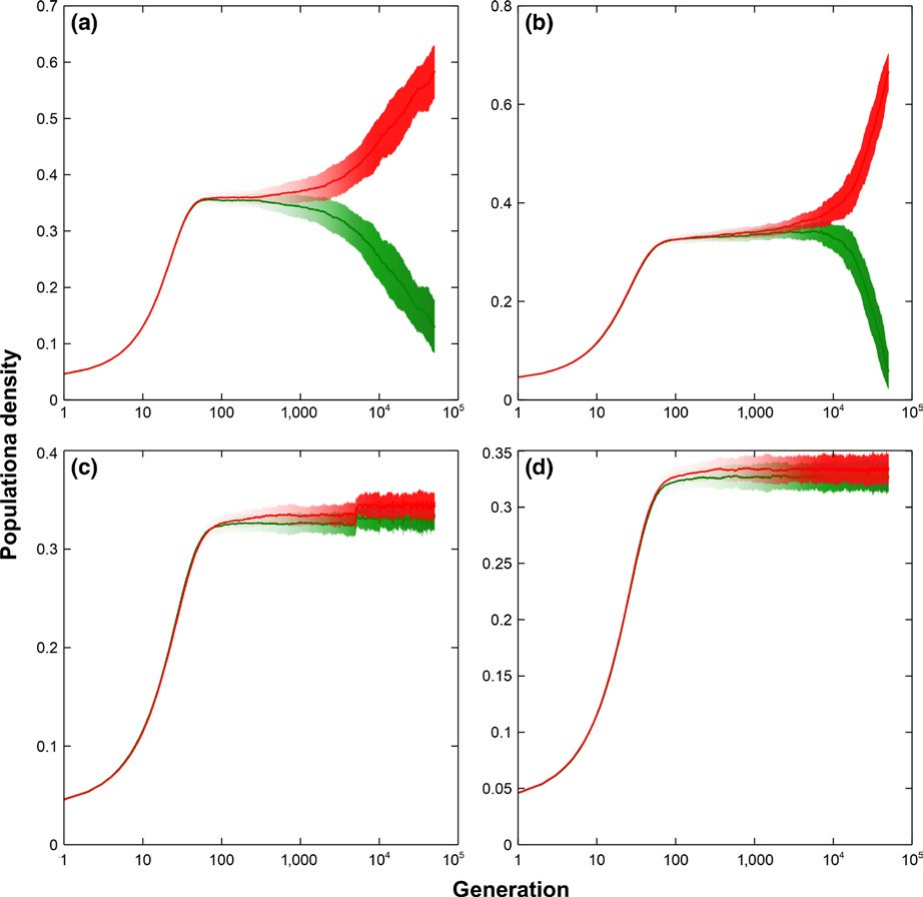
Two‐species dynamics with dependent microhabitat changes under local dispersion. Simulation results of a two‐species lattice Lotka–Volterra competition model with dependent microhabitat locality changes under local dispersion (Average of 50 runs at 50,000 generations with the corresponding standard deviations as error bars). (a) Average population dynamics and deviation of a two‐species system where microhabitat locality changes are large and rate of change is rapid. (b) Average population dynamics and deviation of a two‐species system where microhabitat locality changes are small and rate of change is rapid. (c) Average population dynamics and deviation of a two‐species system where microhabitat locality changes are large and rate of change is slow. (d) Average population dynamics and deviation of a two‐species system where microhabitat locality changes are small and rate of change is slow

**Figure 5:**
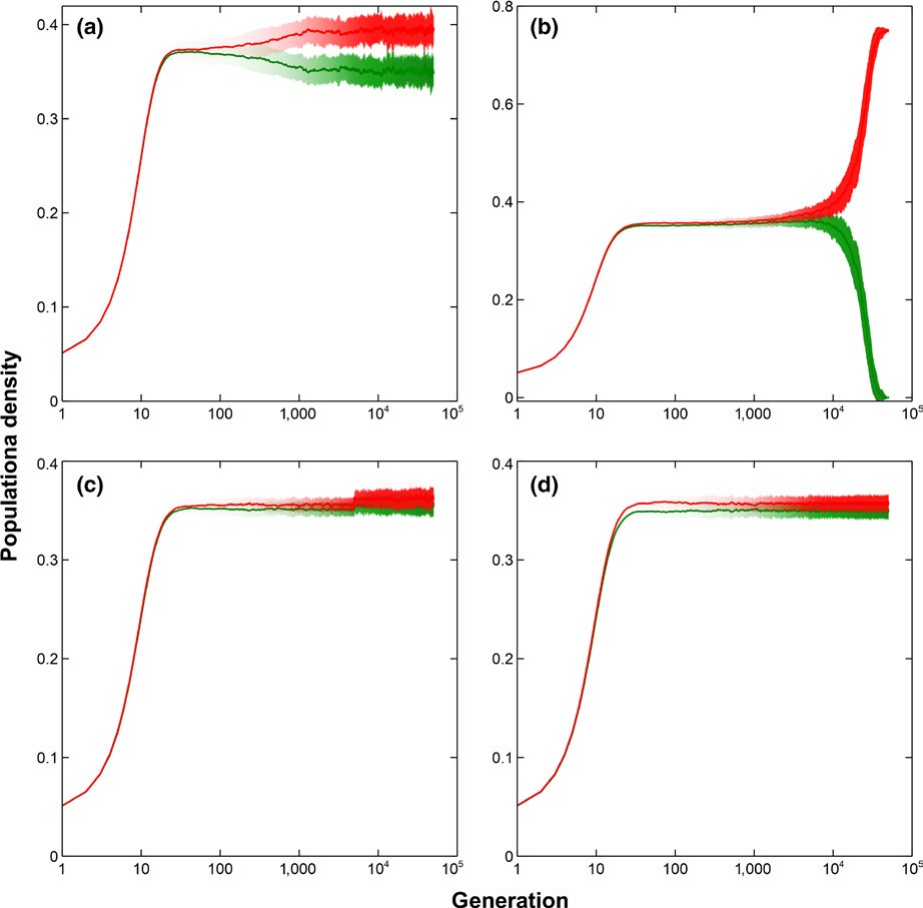
Two‐species dynamics with dependent microhabitat changes under global dispersion. Simulation results of a two‐species lattice Lotka–Volterra competition model with dependent microhabitat locality changes under global dispersion (Average of 50 runs at 50,000 generations with the corresponding standard deviations as error bars). (a) Average population dynamics and deviation of a two‐species system where microhabitat locality changes are large and rate of change is rapid. (b) Average population dynamics and deviation of a two‐species system where microhabitat locality changes are small and rate of change is rapid. (c) Average population dynamics and deviation of a two‐species system where microhabitat locality changes are large and rate of change is slow. (d) Average population dynamics and deviation of a two‐species system where microhabitat locality changes are small and rate of change is slow

To examine the effect of the rate and level of change in microhabitat locality, similar simulations were conducted to a two‐species system. Figures 4 and 5 show the results of the simulation for both local and global dispersion, respectively. Rapid change in microhabitat locality, where the changes happen every generation, leads to exclusion regardless of the level of change when the interaction is local (see Figures 4a,b). For global dispersion, exclusion happens when the rate of change in microhabitat locality is rapid and the level of change is high (see Figure 5b). Although exclusion is avoided when the change is rapid but small, a decreasing trend in the density of one of the species is visible (see Figure 5a). Extending the simulation might lead to exclusion in the long run. For both local and global dispersions, slow change in microhabitat locality, where the changes happen every 5,000 generations, shows relatively stable coexistence (see Figures 4c,d and 5c,d).

### 3.3 Dispersal distance

**Figure 6:**
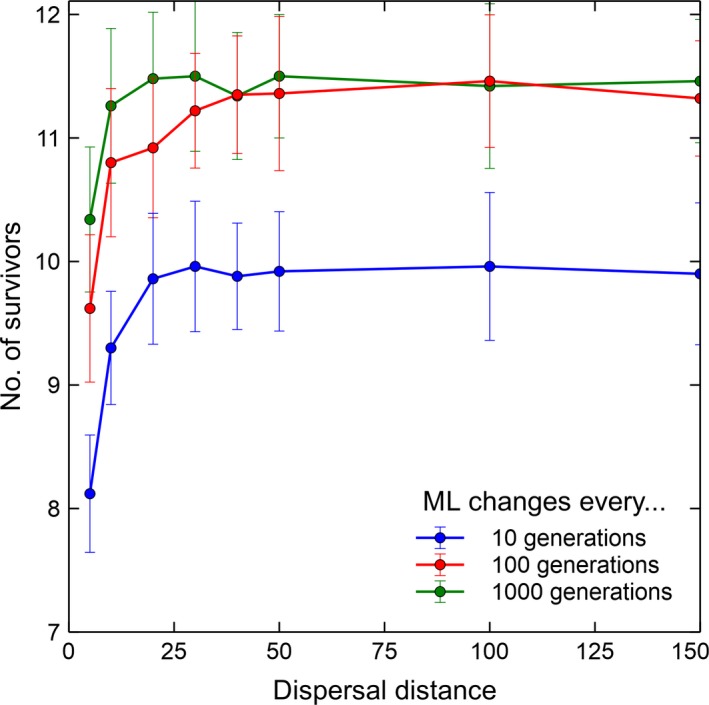
Microhabitat changes and dispersal distance. Temporal changes in the number of surviving species in a 20‐species lattice system with respect to the different dispersal distances and microhabitat locality changes (Average of 50 runs at 50,000 generations with the corresponding standard deviations as error bars)

Effect of dispersal is determined by conducting simulations with different dispersal distance while independent changes in microhabitat locality are in effect. These different dispersal distances are intermediate scenarios compared local and global dispersion. Species can only reproduce to one of the adjacent sites with local interaction while species can disperse anywhere in the lattice in the case of global. Figure 6 shows that the number of coexisting species increases as seedling dispersal increases. In fact, an increase in dispersal distance from one lattice site to 25 increases the number of coexisting species significantly. It is important to note that in our previous study, an increase in dispersal distance has a negative effect to coexistence because it leads to more competition which results in the extinction of weaker species (Tubay et al., 2015). This is in contrast with the results of our new model with a dynamic microhabitat locality. In this model, although longer dispersal distances bring about more competition, they also allow species to find more suitable sites which were formerly not suitable to proliferate avoiding extinction.

Moreover, it demonstrates that slower rate of microhabitat locality change is better for coexistence. A change every one thousand generations allows more species to coexist compared to a change in microhabitat locality every 10 generations. However, at long dispersal distances, the difference between the number of coexisting species between microhabitat locality changes every 100 and 1,000 generations is negligible.

### 3.4 Lattice size

**Figure 7:**
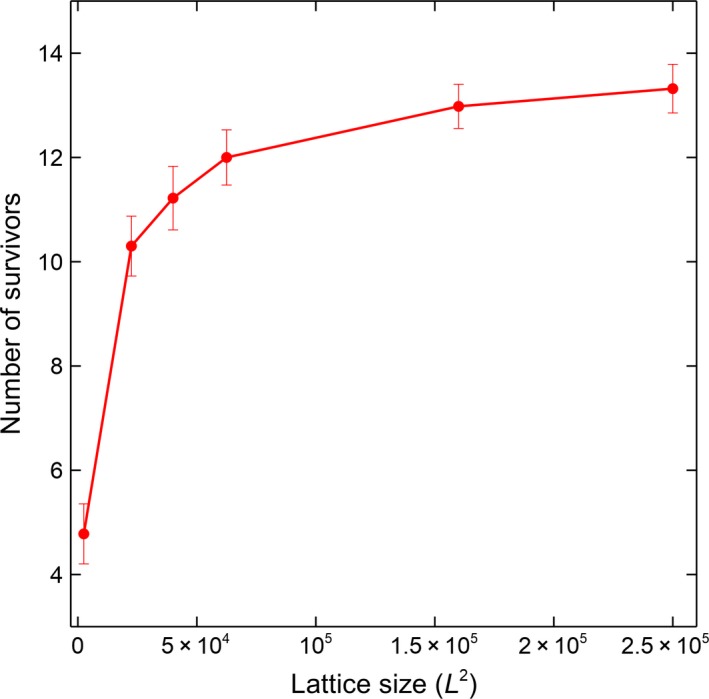
Microhabitat changes and lattice size. Temporal changes in the number of surviving species in a 20‐species lattice system under global dispersion with respect to different lattice sizes where microhabitat locality changes totally in every generation (Average of 50 runs at 50,000 generations with the corresponding standard deviations as error bars)

Similar to the previous lattice coexistence model (Tubay et al., 2015), lattice size or carrying capacity L×L has a positive effect on coexistence. Simulations were conducted using difference lattice sizes from 50 × 50 to 500 × 500 with increments of 50 to L where microhabitat locality changes in every generation. Figure 7 shows that an increase in lattice size also increases the number of surviving species. A significant increase in the number of surviving species can be observed if the lattice size is increased from 50 × 50 to 100 × 100. After the former lattice size, the rate of increase in the number of surviving species decreases.

## 4 DISCUSSION

We have previously shown that microhabitat locality in a plant lattice community guarantees the coexistence of many species (Tubay et al., 2015). The results of this new simulation model show that even dynamic microhabitat locality can still lead to species coexistence. Figure 1 shows that dispersion can neutralize the negative effect of the rapid change in microhabitat locality. However, if the rate of change of microhabitat locality is too fast, the number of coexisting species decreases drastically (see Figure 1b). This only shows that a rapid change in the environment, such as accelerated climate change, has a negative impact on the biodiversity albeit the relatively high resistance of species with long dispersal distances. The risk of competitive exclusion becomes higher as the changes in environment hasten which threatens many vulnerable species with constrained habitat requirements (Bellard et al., 2012).

Looking at the positive side, Figure 2 suggests that as long as microhabitat locality is maintained, notwithstanding its dynamic behavior, coexistence is still possible at least to a two‐species system. This shows that given microhabitat locality is present and the changes are independent of their current values, coexistence is guaranteed. Although coexistence is higher in a 20‐species system if the microhabitat changes are slower and dispersal distances are longer (see Figures 1 and 6). However, the effect of a dynamic microhabitat locality changes drastically when the alterations become dependent.

As stated in the results, gradually changing the microhabitat locality results to a decrease in the number of coexisting species (see Figure 3). In fact, even a very small change can reduce the number of coexisting species radically when the changes occur every generation. However, the unexpected result is that increasing the percentage microhabitat locality change further can lead to a better lattice system for coexistence. The closer the change to a 100 percent, the more species can coexist for both local and global dispersion. In other words, large changes in microhabitat locality promote coexistence, while minute changes do not. This phenomenon can be associated with the relationship between microhabitat locality change and adaptation. Large changes in microhabitat equate to extreme stress which force plant species to adapt. Several studies have shown that accelerated climate change promotes adaptation (Aitken, Yeaman, Holliday, Wang, & Curtis‐McLane, 2008; Davis & Shaw, 2001; Hamrick, 2004). For instance, forest trees have the natural ability to adapt because of their high levels of genetic diversity, longevity, and phenotypic plasticity (Hamrick, 2004). In this model, an adaptation of the species is finding new lattice sites that are currently suitable for them after the change in microhabitats. As the ability to find new suitable sites depends on species’ dispersal distance, the number of coexisting species with a 90 percent change in microhabitat is almost equal to the number of species when the change is almost absent if the dispersion is global (see Figure 3b). The same cannot be said with local dispersion (see Figure 3a), where the number of surviving species at the highest microhabitat change is far smaller compared to when the change is negligible.

In contrast, minute accumulated changes leading to exclusion dampen the response of species to find more suitable sites. As the changes in microhabitat are minuscule, individuals tend to stay and reproduce in the same sites where they previously thrived. However, once the changes accumulated, it becomes too late to adapt especially for weaker species. Evidence of adaptational lag in plants is scarce but it has been recently observed in banked seeds of the annual weed *Arabidopsis thaliana* (Wilczek, Cooper, Korves, & Schmitt, 2014). This lag in adaptation can also be observed in forest trees which is enhanced by longevity especially when change is rapid (Hamrick, 2004). At this point, although a complete change in microhabitat locality is better than that of minute accumulated changes, static microhabitat locality is still best for coexistence.

As we have pointed out previously, longer dispersal distances have a positive effect to coexistence which is not the case in our previous study (see figure 4b of Tubay et al., 2015). This contradiction is possible as a model with a static microhabitat locality is qualitatively different to a dynamic model. Given static microhabitat differences, dispersing to longer means more inter‐specific competition which leads to the exclusion of superior species (Tubay et al., 2015). This happens as superior species establish their territory and proliferate faster than the weaker ones. However, this is not the case when microhabitat differences are changing. In this current model, establishing the population becomes harder to even superior species as site suitability continues to change particularly when the interval between changes is short.

Unlike the result with dispersal distance, the effect of lattice size or carrying capacity to coexistence when microhabitat locality is dynamic is similar to the old model. This is expected as more lattice sites mean more spaces to proliferate even for weaker species. The increase in the number of surviving species in the smaller lattice sizes is faster compared to the larger ones as the increase in the number of lattice sites is greater (i.e., 50 × 50 = 2, 500 to 100 × 100 = 10, 000 (quadrupled) compared to 100 × 100 = 10, 000 to 150 × 150 = 22, 500 (more than doubled only)).

Microhabitat locality allows species to coexist because trade‐offs are preserved, providing each of the different species space where they are advantageous. This situation permits them to thrive which weaken interspecies competition. However, modifying this minute differences in microhabitat continually in regular time intervals leads to more competition. They are competing to adapt and the ones that can find new suitable sites become winners. Static microhabitat locality promotes competition avoidance while dynamic differences force different species to adapt and compete.

Now, it is very important to emphasize that the primary driver for species extinction is the speed by which microhabitat is changing particularly for global dispersion (see Figures 3, 4 and 5). Although the level of change can affect coexistence, it is clear from Figures 3, 4, and 5 that no matter how small or big the change is, if the species cannot keep up with the speed of the change, exclusion is inevitable. A perfect example is the speed by which the climate is currently changing and its impact to the biodiversity (Gitay, Suárez, Watson, & Dokken, 2002; Thompson, 2010). According to Thompson, we only have three options for our inaction: mitigation, adaptation, or suffer the consequences.

Ultimately, this new model captures the basic principle for the coexistence and exclusion of sessile species in a changing environment which has been an important issue due to the effects of climate change. A changing climate is normal and has been happening since the birth of our planet. However, what is unusual is its current speed. This model shows that if the gradual change in microhabitat happens in a speed by which species cannot respond by means of adaptation, extinction will be the only option, especially for vulnerable species.

## CONFLICT OF INTEREST

None declared.

## AUTHOR CONTRIBUTIONS

J. M. T. and J. Y. conceived the study, J. M. T. built the model and conducted the simulations. Finally, both J. M. T. and J. Y. interpreted the results and wrote the manuscript.
